# Internet-based interpersonal psychotherapy for stress, anxiety, and depression in prenatal women: study protocol for a pilot randomized controlled trial

**DOI:** 10.1186/s13063-019-3897-z

**Published:** 2019-12-30

**Authors:** Katherine S. Bright, Muhammad Kashif Mughal, Abdul Wajid, Marie Lane-Smith, Lindsay Murray, Nicola Roy, Sander Veldhuyzen Van Zanten, Deborah A. Mcneil, Scott Stuart, Dawn Kingston

**Affiliations:** 1 Faculty of Nursing, University of Calgary, 2500 University Dr. N.W, Calgary, AB T2N 1N4 Canada; 2 Department of Medicine, Division of Gastroenterology, University of Alberta, Edmonton, AB T6G 2R7 Canada; 3 Alberta Health Services, Scientific Director, Maternal Newborn Child and Youth Strategic Clinical Network, Southport Atrium #2237, 10101 Southport Road, S.W., Calgary, AB T2W 3N2 Canada; 4 Interpersonal Psychotherapy (IPT) Institute, PO Box 5925, Coralville, Iowa 52241 USA

**Keywords:** Perinatal women, Interpersonal psychotherapy, Psychological distress, Mental health, Internet, Online, Depression, Stress, Anxiety

## Abstract

**Background:**

Psychological distress, defined as depression, anxiety and perceived stress, during pregnancy is common, with 15–25% of women experiencing clinically significant levels of such distress. Despite the far-reaching impact of prenatal psychological distress on mothers and their children, and that women are receptive to screening, few providers routinely screen for prenatal psychological distress and less than one in five women will receive the mental health care that they require. There is a lack of certainty regarding the most effective treatments for prenatal psychological distress. No online interpersonal psychotherapy (IPT) trials have been conducted that focus on improving psychological distress in prenatal women. The purpose of this pilot randomized controlled trial is to evaluate the perspectives of pregnant women on the feasibility and acceptability of online IPT (e-IPT) delivered during pregnancy.

**Methods:**

A pilot randomized controlled trial design with repeated measures will evaluate the feasibility and acceptability of e-IPT for pregnant women compared to routine prenatal care. Qualitative interviews with 15–30 individuals in the intervention group will provide further data on the feasibility and acceptability of the intervention. Assessment of feasibility will include the ease of accessing and completing the intervention. Women will also be asked about what barriers there were to starting and completing the e-IPT. Assessment of acceptability will inquire about the perception of women regarding the intervention and its various features. A sample size of 160 consenting pregnant women aged 18 years and older will be enrolled and randomized into the experimental (e-IPT) or control (routine care) condition. The secondary outcome measures include: depression, anxiety and stress symptoms; self-efficacy; self-mastery; self-esteem; relationship quality (spouse, immediate family members); coping; and resilience. All participants will complete the aforementioned measures at baseline during pregnancy (T_1_), 3 months postrandomization (T_2_), at 8 months of pregnancy (T_3_), and 3 months postpartum (T_4_).

**Discussion:**

The results of this pilot randomized controlled trial will provide data on the feasibility and acceptability of the intervention and identify necessary adaptations. This study will allow for optimization of full trial processes and inform the evaluation strategy, including sample size calculations for the full randomized controlled trial.

**Trial registration:**

ClinicalTrials.gov, NCT01901796. Registered on 18 December 2014.

## Background

### Prenatal psychological distress

The perinatal period is a time of increased vulnerability where women experience greater probability of psychological distress [1, 2]. Psychological distress during pregnancy is common, with 15–25% of women experiencing clinically significant levels of depression, anxiety and stress [3]. While affective disorders have frequently been attributed to hormonal changes [4], the greatest risk factors include modifiable lifestyle factors, including the partner relationship and social support [5]. Left untreated, prenatal psychological distress is associated with poor obstetrical outcomes and maternal functioning, increased incidence of poor child development and cognitive delays, interpersonal conflict, parenting stress, and maternal postpartum mood disorders [6–9].

### Under-detection and under-treatment of prenatal psychological distress

Prenatal maternal psychological distress has a significant and far reaching impact on mothers and their children [6]. There are well-established recommendations for routine mental health screening during the perinatal period [10]. Unfortunately, less than one in five providers routinely screen for perinatal mental health concerns [11]. Equally concerning is that only one in seven perinatal women will receive the mental health intervention they need [11, 12].

There is a lack of certainty regarding the most effective treatments for prenatal psychological distress. The US Preventive Services Task Force completed a systematic evidence review on interventions to prevent perinatal depression [13]. Of the 20 counseling intervention studies included, most using cognitive behavioral therapy (CBT) or interpersonal psychotherapy (IPT), half delivered the intervention to pregnant women and half to postpartum women in the USA [13]. Across these interventions, absolute reductions in risk of depression ranged from 1.3% greater reduction in the control group to 31.8% in the intervention group [13]. No harms, potential or actual, of the interventions were reported. Acceptability of the intervention was reported as positive in several of the studies (*n* = 6) and participants felt that the interventions were beneficial and enjoyable [13]. This review indicated that interventions are effective when delivered during pregnancy, the postpartum period, or both.

These US Preventive Services Task Force findings are similar to another recent literature review that endorsed the moderate treatment effect and lasting benefits of CBT and IPT for pregnant women with a major depressive disorder [14]. Both systematic literature reviews and the US Preventive Services Task Force recommendation statement call for future research on treatments for all mental disorders during pregnancy and larger scale effectiveness trials of CBT and IPT [13–15]. Additionally, significant health system-level limitations in accessing therapies should be addressed [15–17]. In addition to a lack of perinatal-specific training/resources (differentiating between prenatal and postpartum intervention), and non-baby-friendly environments, in-person providers cannot meet the needs of women during the perinatal period [17].

### Interpersonal psychotherapy: aim and effectiveness

The aim of IPT is to improve social support networks since enhanced social support predicts greater psychological adjustment to stressors; IPT is therefore a promising intervention for prenatal women [18–21]. IPT is a short-term dynamic attachment-based psychotherapy [22, 23] aimed at symptom reduction and improved interpersonal relationships through enhanced social support [23]. The primary foci of treatment with IPT are the interpersonal issues of grief and loss, interpersonal disputes, and role transitions (Stuart, 2012). IPT attempts to provide clarification of individuals’ feelings, expectations, interactions, social roles, education, and social competence [24]. Additionally, IPT practitioners endeavor to assist individuals in establishing or better mobilizing support networks [23]. Individual functioning is believed to be based on a combination of intrapersonal factors (temperament, personality and attachment style) in relation to interpersonal factors (social support and quality of relationships) [23].

IPT is a highly effective treatment for depression and anxiety [25–30]. Randomized controlled trials of IPT in perinatal women found that IPT improved symptoms of depression and anxiety [20, 31–35] and functioning [9, 36]. The US Preventive Services Task Force showed convincing evidence that IPT is effective in preventing perinatal depression [13]. A 2011 meta-analysis found that IPT was more effective than CBT for treating perinatal depression [36]. A 2011 meta-analysis also found that IPT was more effective than couples therapy in the treatment of depressive symptoms during the perinatal period [37].

Automatic internet-based therapy interventions offer access to psychological resources at reduced costs, can be accessed from across the world, and can be accessed at any time. Research supporting the use of internet interventions for the prevention and treatment of perinatal mental health concerns is limited but several trials have demonstrated preliminary effectiveness in the prevention [38–40] and treatment of postpartum depression [41–43]. The recommendations from the gaps in the literature leads towards a trial of online IPT (e-IPT). To date, no trials have focused on improving psychological distress in prenatal women as a result of receiving e-IPT.

### Predictors and moderators of response to internet-based psychotherapy

CBT and IPT are reported to have similar effectiveness in reducing depressive symptoms [44–47]. Donker et al. concluded that, even when treatments are determined equally effective, there are individual characteristics (gender, age, education level, marital status, baseline depression level, coping skills, and previous depression) that influence treatment outcomes [28]. Individual characteristics that influence treatment effectiveness can be broken down into predictors and moderators [28]. Both predictors and moderators are considered pretreatment variables; however, the predictors forecast mental health outcomes in the intervention groups, while moderators identify persons more likely to benefit from particular treatments [28]. Examples of predictors of the efficacy of internet-based interventions include whether the internet-based therapies (CBT, IPT) are guided [28, 48], being female, obtaining low mastery scores on self-mastery measures and low dysfunctional attitudes on coping questionnaires [28]. A moderator of internet-based therapy is age, with evidence indicating that older adults benefit from CBT and younger adults demonstrate larger improvements with IPT [28]. A moderator of IPT is also a role transition, with role transitions occurring in younger adults of childbearing years [28]. Individuals undergoing role transitions are thought to benefit more from internet-based IPT because of the foci of IPT; specifically, interpersonal conflicts and role transitions are particularly relevant in their stage of life [28, 49]. As a result, it is expected that younger adults navigating a significant role transition (e.g., being pregnant and becoming a mother) would benefit from guided, internet-based IPT to improve symptoms of depression, anxiety, stress and interpersonal functioning [50].

### The challenge of IPT

IPT is an intervention endorsed as being effective for perinatal women with a history of depression and for individuals currently experiencing depressive symptoms [13]. IPT contains specific components such as developing a support system, effective communication, and skills to manage conflict in relationships for women during the perinatal period [13]. Despite IPT being an effective treatment for perinatal women, access to face-to-face IPT remains a challenge due to there being few trained IPT therapists, long wait times to access care, and high costs to access therapy [51, 52].

There is a need to make effective interventions more available and accessible for pregnant women. Online psychotherapy is an ideal treatment option for individuals as it overcomes major obstacles to psychological support including long wait times, lack of time, the stigma of accessing care, and financial burden [51]. In addition to higher levels of patient adherence and convincing reductions in mental health symptoms, online treatment options are readily available and cost-effective options to face-to-fact treatment [52]. Additionally, online psychological treatments are more effective and significantly less expensive than face-to-face therapy, are stable in the short and long term, and adherence rates are 80% [53]. In a meta-analysis of online psychotherapy, the advantages of online therapy included convenience, ability for individuals to proceed at their own pace, low cost, no wait times, high effect size, and superior attendance compared to face-to-face or group therapy [52]. Our study addresses the challenge of face-to-face IPT by evaluating online delivery of IPT.

### Research objectives, questions, and hypotheses

The primary aim of this pilot randomized controlled trial is to examine feasibility and acceptibility of e-IPT for prenatal women. The assessment of feasibility will include the percentage of women in the intervention group who report that the e-IPT is integrated as a component of prenatal care without difficulty. Acceptability will be assessed in terms of the percentage of women in the intervention group who report that modules and activities in e-IPT are easily understood and navigated. The primary research objective, question, and hypothesis are described in Table 1. The secondary objectives include assessment of symptoms of anxiety, depression, overall psychological distress, relationship quality (spouse, immediate family members), and mother resilience compared to routine care. We hypothesize that the e-IPT intervention will reduce symptoms of distress, anxiety and depression and will be significanly more effective compared to usual care. These secondary research objectives, questions, and hypotheses are described in Table 2.

**Table 1:** Primary objective, research questions, and hypothesis

	Objective	Research questions	Testable hypothesis
1	To assess the feasibility and acceptability of online interpersonal psychotherapy for prenatal women	Do prenatal women find the online IPT intervention is easily integrated as a component of prenatal care without difficulty? Do prenatal women find the online IPT intervention acceptable? What will improve the performance of online interpersonal psychotherapy in a full trial version for prenatal women? Is it feasible to enroll and retain prenatal women in a study to assess an online IPT intervention? Is it feasible to perform data collection procedures as planned? Is it feasible to deliver the IPT program as intended?	Prenatal women will define strategies to enhance online interpersonal psychotherapy, readying it for a full trial

*IPT* interpersonal psychotherapy

**Table 2:** Secondary objectives, research questions, and hypotheses

	Objective	Research question	Testable hypotheses
1	To evaluate the clinical effectiveness of online interpersonal psychotherapy versus routine care on reducing stress, anxiety, and depression in prenatal women	What is the effect of an online interpersonal psychotherapy intervention compared to routine care on the percentage of prenatal women with symptoms of stress, anxiety, and depression?	The percentage of prenatal women with stress, anxiety and depression about the clinical cut-offs of validated tools will be significantly lower in the intervention group versus routine care
2	To evaluate the effectiveness of the online interpersonal psychotherapy intervention compared to routine prenatal care on relationship distress	Is relationship distress reduced in the online interpersonal psychotherapy intervention group compared to the routine prenatal care control group?	Relationship distress will be significantly reduced in the online interpersonal psychotherapy group
3	To identify factors, mediators and moderators that are associated with effectiveness of the intervention among participants within the intervention group	What factors, mediators, and moderators are associated with the intervention effect?	Among participants in the intervention group, no factors will be significantly associated with the intervention effect, indicating that online interpersonal psychotherapy is appropriate for prenatal women

## Methods/design

**Fig. 1.**
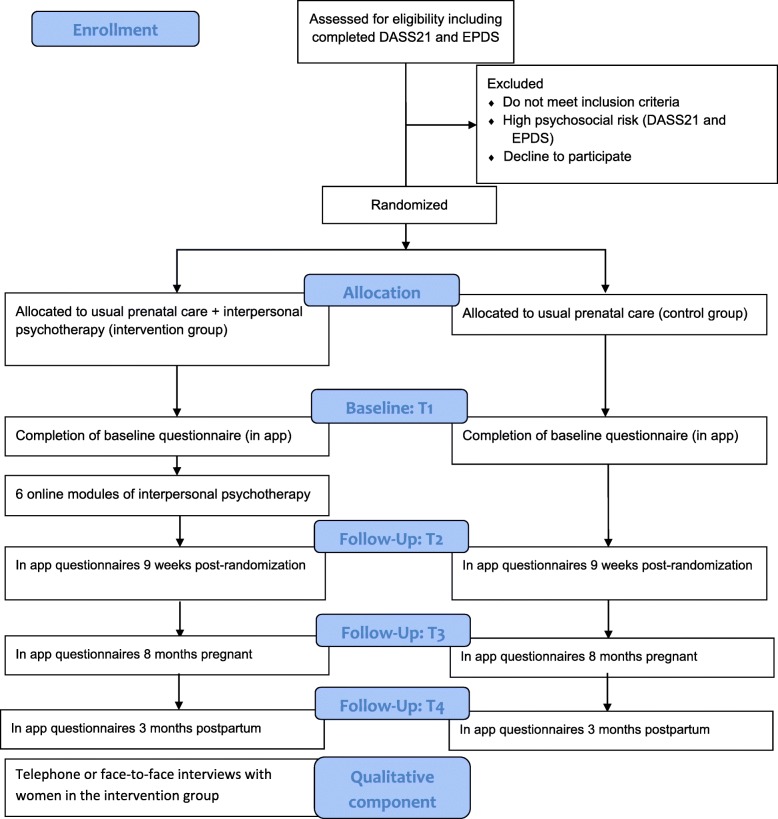
SPIRIT schedule of enrollment, interventions, and assessments

This protocol is reported according to the Standard Protocol Items: Recommendations for Interventional Trials guidelines (Fig. 1).

### Study design

**Fig. 2.**
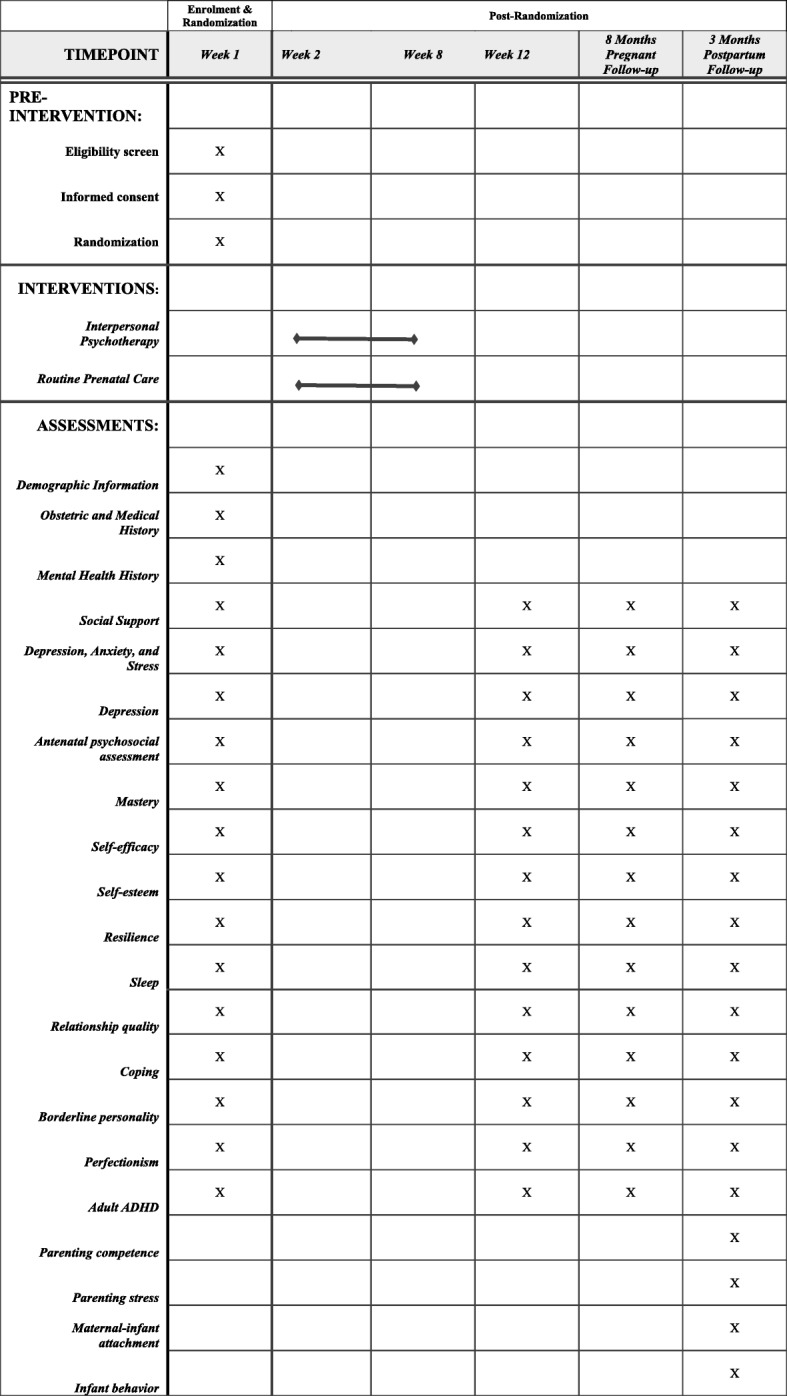
Consolidated Standards of Reporting Trials 2010 flow diagram. DASS21 Depression, Anxiety, and Stress Scale-21, EPDS Edinburgh Postnatal Depression Scale

This pilot study is a randomized controlled trial of two parallel groups. The intention of this study is to evaluate the feasibility and acceptability of IPT in a online format for a sample of prenatal women. Additionally, this study is a parallel group, Consolidated Standards of Reporting Trials revised 2010 compliant, pilot randomized controlled trial of e-IPT versus routine prenatal care (Fig. 2). Data collection points are at baseline, 3 months postrandomization, and at 3 and 6 months postpartum. The current trial protocol has been approved by the Conjoint Health Research Ethics Board at the University of Calgary (REB16–0061). This study is registered with ClinicalTrials.gov (identifier NCT1901796).

### Participants

Pregnant women will be recruited through established recruitment sources including maternity clinics, posted announcements and pamphlets available in the main areas of family medical clinics and other areas that pregnant women frequent (e.g., health centers, antenatal clinics, prenatal classes, hospitals). Participants will also be recruited through online advertising and media outreach.

Health-care providers will ask eligible pregnant women if they would like to participate in this study and will provide these women with a study postcard containing the link to the online study web application. The study postcard will also contain contact information for the research coordinator who is available to answer any outstanding questions for participants.

### Inclusion and exclusion criteria

Eligible participants are women who reside in Alberta, Canada, are over the age of 18 years and are pregnant, from conception to 30 weeks gestation. Participants must be able to read and write in English. Participants must be capable of providing informed consent and of learning and understanding new information such as contained in the e-IPT modules. Additionally, participants must have access to online hardware including a digital device (either a computer, smart phone, or tablet).

### Screening, consent, and enrollment

Study postcards containing the link to the online study web application will be provided to potential participants. The link to the online study web application will describe the nature of the study, the study protocol, and randomization procedures. If participants are interested, they will electronically consent to participate by responding affirmatively to a single-step consent process on the online consent form. Participants will also be contacted by email and asked if they would consent to a telephone interview about the acceptability of the intervention after they have completed the 3-month postpartum questionnaire.

### Intervention: online interpersonal psychotherapy and telephone coach

Participants in the intervention group will be asked to complete six, 30-min online interactive IPT modules over a 6-week period. Modules will be embedded in an online app that participants access with a personalized login username and password. The topics covered in the modules are: 1) identifying the important relationships in their life; 2) understanding and improving communication patterns; 3) navigating interpersonal disputes; 4) adapting to role transitions; 5) working through grief and loss; and 6) maintaining IPT strategies following study completion. These topics were designed based on the work of Stuart and Robertson and tailored to pregnancy [23]. Participants are asked to complete specific assignments online for each module such as self-awareness homework, exploring relationships with those closest to you. Goals of the intervention include symptom relief [54], improving interpersonal functioning and relationships [22, 23], changing expectations about interpersonal relationships [49], and improving participant social support networks [23]. Participants are guided to recognize and disengage from unhelpful communication patterns and develop strategies for engaging social supports in navigating challenging times such as grief and loss, role transitions, and conflict with their partner or extended family members [55].

Participants in the intervention group will receive psychosocial assessments using the questionnaires outlined in Fig. 1. These questionnaires will be administered at 3 months postrandomization and again at 3 and 6 months postpartum.

Telephone coaches will be available to the intervention group at any time; however, the coaches also contact women under specific conditions, including having very high symptoms of depression, anxiety or stress, experiencing a pregnancy loss during their study participation, or having complex needs (e.g., high psychological and social needs). Telephone coaches consist of a social worker and a nurse who have identical formal IPT, suicide assessment and prevention, interviewing, and counseling training. Coaches are supervised weekly to address and maintain fidelity in the study protocol.

### Control condition: routine prenatal care

Participants in the control group will receive routine prenatal care which usually does not include standardized psychosocial assessment and, in most cases, treatment. Women in the control group of this study will receive psychosocial assessments using the same questionnaires at the same time points as those administered to the intervention group.

### Both groups

All applicants will complete the Depression, Anxiety, and Stress Scale-21 (DASS21) and the Edinburgh Postnatal Depression Scale (EPDS) on recruitment (prerandomization) to provide baseline data on their mental health status. All participants (control and intervention groups) assessed as having thoughts of self-harm will receive a computerized message offering then crisis line/emergency room contact information and informing them that they will be contacted by the study nurse. Following randomization, participants will complete the baseline series of questionnaires which comprehensively assess a variety of demographics and psychosocial constructs (T_1_) (Fig. 1). These questionnaires will be completed again at 3 months postrandomization (T_2_), after 8 months of pregnancy (T_3_), and 3 months postpartum (T_4_).

### Methods to protect against bias: randomization and blinding

Eligible women will complete the consent to participate in the online app and will then be automatically randomized within the app at a 1:1 control to treatment ratio. Immediately following randomization women will be notified of their group assignment through the app. Participants are not blind to their group allocation, nor are their health-care providers who are informed of the screening results of their patients (if the participant consents to fax a report to her health-care provider) and recommendation for e-IPT.

### Definition and measurement of outcomes

#### Primary outcomes

The primary outcome of this pilot study is the feasibility and acceptability of e-IPT for pregnant women. Feasibility of the e-IPT intervention will also include an examination of enrollment and retention of women in the study. Additionally, the assessment of feasibility will assess adherence to the planned methods of data collection and intervention delivery. The feasibility interviews with 30 participants in the intervention group will ask women to comment on how easy it was to access and complete the intervention, as well as how long it took to complete the modules. In this study we also define feasibility as the percentage of women in the intervention group who report that the e-IPT intervention is easily done as a component of care during the perinatal period. Women in the e-IPT intervention group will also answer questions rating their experiences of the intervention. Women in the intervention group will answer questions to assess their views of e-IPT (for example, ‘were you comfortable using e-IPT with an online coach for therapy?’). Women in the intervention group will also be asked about suggestions for improving the e-IPT.

In this study we define acceptability as the percentage of participants in the intervention group who report that e-IPT is acceptable, the percentage of participants reporting that the e-IPT modules were easy to understand and navigate, the percentage of participants who found that the information in the module was effective in achieving intended outcomes, and whether the exercise was clear and easy to understand. Additionally, participants in the intervention group will be asked whether they would recommend the module to a pregnant friend. Women receiving the e-IPT will also be asked several questions to evaluate their user experience with the modules.

#### Strategies for assessing adherence and attrition

To assess adherence, the completion of online questionnaires and the intervention homework will be recorded through a specific application through Google Analytics. This application will include the length of time taken to complete modules and the number of modules completed. To assess attrition, comparisons between the attrition rates of the control and the intervention groups will be performed.

#### Secondary outcomes

##### Psychological outcomes

Secondary outcomes of this pilot study evaluate the effectiveness of e-IPT compared to routine prenatal care with respect to a variety of intraindividual and interpersonal outcomes (Table 2), specifically stress, anxiety, depression, and relationship quality in prenatal women. The DASS21 is a 21-item questionnaire used to examine three self-report subscales including depression, anxiety, and stress over the previous week. Responses are recorded on a Likert-type scale ranging from 0 (not applicable at all) to 3 (applicable very much of the time) [56]. Based on the scores of each domain (raw scores multiplied by 2) the severity is graded into normal, mild, moderate, severe, or extremely severe [56]. The DASS21 is a well-validated (concurrent validity coefficients were 0.87 and 0.84) and reliable (test–retest and split-half reliability coefficient scores of 0.99 and 0.96) questionnaire [57] that has been previously used in perinatal samples [58–61]. The EPDS will be used to assess symptoms of depression experienced during pregnancy and the postpartum period. The EPDS is a well-validated questionnaire that has been previously used in pregnant women [62–65]. The EPDS is a 10-item self-report questionnaire used to assess perinatal depression (eight items) and anxiety (two items) over the previous week [66]. The questions are a Likert scale scored from 0 to 3 (with every changing anchors) with higher scores indicating more depressive symptoms. The EPDS is well validated, with sensitivity and specificity of 86% and 78%, respectively [67]. There is also good reliability for the EPDS, with test–retest reliability of 0.92 [68]. Additional secondary outcomes will be evaluated including relationship quality, resiliency, maternal–infant attachment, infant behavior, and parenting stress/competence.

##### Variables

Variables that are associated with (and may mediate) the effectiveness of the e-IPT will be explored through an examination of variables that may be influential in the perinatal mental health theories and literature. The factors to be included were selected based on their association with perinatal depression, anxiety and stress. As a result, the influence of self-efficacy, social support, sense of mastery, self-esteem, sleep, coping, and resilience will be explored. Additionally, the association of preconception of mental health problems such as borderline personality disorder, perfectionism, and attention deficit/hyperactivity disorder will be investigated. These variables will be included as covariates in the data analysis models.

### Demographic assessment

Demographic information will include marital status, number of children in the home, ethnicity, age, family socioeconomic status (household income, education level) and working status. Assessment of previous mental health problems and assessment of mental health treatment (medication or counseling) will be assessed.

### Risks to the safety of participants

#### Intervention group

At the end of each e-IPT module participants will complete question 10 of the EPDS assessing thoughts of self-harm over the last 7 days. A response of anything other than ‘never’ will initiate the study nurse to contact the participant within 24 h.

#### Both groups

Participants who decompensate in their mental health during the course of the study will be able to request mental health support from the study coaches within the online web application. Once this request has been triggered in the system, a study coach will contact the participant within 24 h. If any adverse events occur (increased suicidal ideation, converting from no risk to low or moderate risk) the study nurse has a treatment algorithm in place to triage participants to appropriate acute or long-term treatments, as needed, to ensure the participant’s safety. Additionally, adverse events will be reported to the University of Calgary Ethics Review Board.

### Sample size estimation

The DASS21 categorizes women as having normal, mild, moderate, severe, and extremely severe depression, anxiety and stress [56]. To shift women from ‘midrange’ moderate symptoms down to mild severity on the depression, anxiety and stress subscales, these women would require a reduction of four points in each subscale. Based on the sample size formula for comparison of two means (two-tailed) at a significance level of 5% (1.96), a power of 80% (0.84), and a minimally clinically important difference of four points, 204 women with mild to moderate psychological distress are required in the trial. Based on a 25% prevalence rate of low–moderate prenatal psychological distress [69–71], a final sample size of 816 eligible women (408 per group) would be needed. This corresponds to a moderate effect size (*d* = 0.50) for depression, which is consistent with a meta-analysis of effect sizes of e-IPT [25]. Therefore, this final sample size of 816 participants is adequate to address the primary and secondary research questions of a full trial. Accounting for a participation rate of 50% based on previous studies of psychotherapy in pregnant women [55, 72, 73], the exclusion of 15% of women who do not meet study criteria (5% high psychosocial risk [70, 71, 74], 10% non-English speaking women), an attrition rate of 25% based on previous studies of perinatal IPT [20, 55], and a 5% loss to follow-up, 1592 women would need to be invited to participate in the study to achieve the final full trial sample size. As this is a pilot study, obtaining a sample size of 10% of the full trial sample is sufficient [75]; therefore 160 participants will be included in the pilot study with 80 participants in the intervention and control groups, respectively.

### Data collection

There are three data collection points for both intervention and control groups: recruitment (T_1_), 3 months postrandomization (T_2_), after 8 months of pregnancy (T_3_), and 3 months postpartum (T_4_)_._ (Fig. 1). All questionnaires will be completed online though a web application. Participants will receive computer-generated emails or smartphone reminders to complete questionnaires. These reminders will occur at 1, 3, and 7 weeks.

In addition to the online questionnaires and online modules, participants in the intervention group will be asked to participate in interviews regarding the feasibility and acceptability of the intervention. Additionally, women will be asked about suggestions for improving the e-IPT. Thirty women in the intervention group will complete the interviews. Using a semistructured interview schedule, these women will be asked about their experiences with the online intervention. These interviews will be transcribed verbatim and then coded and analyzed using an inductive thematic analysis approach [76].

### Data analyses

#### Feasibility and acceptability of the intervention

Feasibility and acceptability, in addition to the qualitative data obtained from the interviews, will be characterized using descriptive statistics (frequencies, proportions, means, standard deviations). Feasibility will be assessed as the rate of retention in the study and percentage of modules completed. The acceptability of this intervention will consist of an analysis of the perceptions of the prenatal women regarding the intervention and its various features. Acceptability will be assessed by open-ended questions exploring the reported ease for women in navigating the online intervention. Participants will also be asked about what was useful and that was difficult in navigating this online intervention. The qualitative data will be analyzed using inductive thematic analysis. This method was chosen because it allows patterns and key themes within the data to be identified [76]. The three-stage process of thematic analysis will involve: 1) generating code; 2) merging and renaming similar codes; and 3) grouping codes into overarching themes that best describe the data [77].

#### Quantitative analysis

The quantitative dataset will be analyzed using IBM SPSS Statistics software. Missing values will be managed by using multiple imputation [78]. Assessment of the presence of any outliers will be made, and it will be determined if they are true values or data entry errors [78]. If not determined as an entry error, the analysis will be run with the outliers included and then run without the outliers included. Results will be compared and whether the outliers skew the results and what it means on a clinical level will be reported. If the results do not differ, then the analysis with the outliers included will be used. Outcomes will be assessed based on the number of modules completed. Additionally, a sensitivity analysis will be completed by running a negative binomial regression model which accounts for variance in the data. Descriptive statistics (mean/standard deviation or frequency/proportions) will serve to describe the characteristics of participants and assess group equivalence at baseline. Repeated measures analysis of variance will be used to determine the effectiveness of the intervention. The variables will be included in the analysis as covariates and bivariate analysis will be performed first. Those variables found to be statistically significant at the bivariate level will be included in the multivariate model. Analysis will adhere to the intent-to-treat principle [78].

### Management

DK will be responsible for overall project management.

### Trial status

This study, protocol version V.1, dated 24 May 2019, commenced recruitment in March 2019. This pilot study will be performed over a 6-month period. We will disseminate trial results through research articles.

## Data Availability

Participants will complete questionnaires online and these data will be sent to a secure Canadian Amazon server. There the data will be anonymized and then downloaded to a secure server at the Health Research Data Repository (HRDR), housed in the Faculty of Nursing, University of Alberta. Additionally, the data will be stored and analyzed within the HRDR. HRDR access is restricted to the research team conducting data analyses. Qualitative interviews will be digitally recorded and transcribed verbatim. These transcribed interviews and digital files will be password protected and stored on a password-protected computer in a locked, secure office at the University of Calgary. We expect to complete feasibility and acceptability interviews by October 2019.

## Abbreviations

CBT: Cognitive behavioral therapy
DASS21: Depression, Anxiety, and Stress Scale-21
e-IPT: Online interpersonal psychotherapy
EPDS: Edinburgh Postnatal Depression Scale
IPT: Interpersonal psychotherapy
